# TspanC8 Tetraspanins and A Disintegrin and Metalloprotease 10 (ADAM10) Interact via Their Extracellular Regions: EVIDENCE FOR DISTINCT BINDING MECHANISMS FOR DIFFERENT TspanC8 PROTEINS*

**DOI:** 10.1074/jbc.M115.703058

**Published:** 2015-12-14

**Authors:** Peter J. Noy, Jing Yang, Jasmeet S. Reyat, Alexandra L. Matthews, Alice E. Charlton, Joanna Furmston, David A. Rogers, G. Ed Rainger, Michael G. Tomlinson

**Affiliations:** From the ‡ School of Biosciences, College of Life and Environmental Sciences, University of Birmingham, Birmingham B15 2TT, United Kingdom and; § School of Clinical and Experimental Medicine, College of Medical and Dental Sciences, University of Birmingham, Birmingham B15 2TT, United Kingdom

**Keywords:** ADAM, cell surface enzyme, endothelial cell, metalloprotease, N-cadherin, platelet, shedding, tetraspanin, GPVI, TspanC8

## Abstract

A disintegrin and metalloprotease 10 (ADAM10) is a ubiquitously expressed transmembrane metalloprotease that cleaves the extracellular regions from its transmembrane substrates. ADAM10 is essential for embryonic development and is implicated in cancer, Alzheimer, and inflammatory diseases. The tetraspanins are a superfamily of 33 four-transmembrane proteins in mammals, of which the TspanC8 subgroup (Tspan5, 10, 14, 15, 17, and 33) promote ADAM10 intracellular trafficking and enzymatic maturation. However, the interaction between TspanC8s and ADAM10 has only been demonstrated in overexpression systems and the interaction mechanism remains undefined. To address these issues, an antibody was developed to Tspan14, which was used to show co-immunoprecipitation of Tspan14 with ADAM10 in primary human cells. Chimeric Tspan14 constructs demonstrated that the large extracellular loop of Tspan14 mediated its co-immunoprecipitation with ADAM10, and promoted ADAM10 maturation and trafficking to the cell surface. Chimeric ADAM10 constructs showed that membrane-proximal stalk, cysteine-rich, and disintegrin domains of ADAM10 mediated its co-immunoprecipitation with Tspan14 and other TspanC8s. This TspanC8-interacting region was required for ADAM10 exit from the endoplasmic reticulum. Truncated ADAM10 constructs revealed differential TspanC8 binding requirements for the stalk, cysteine-rich, and disintegrin domains. Moreover, Tspan15was the only TspanC8 to promote cleavage of the ADAM10 substrate N-cadherin, whereas Tspan14 was unique in reducing cleavage of the platelet collagen receptor GPVI. These findings suggest that ADAM10 may adopt distinct conformations in complex with different TspanC8s, which could impact on substrate selectivity. Furthermore, this study identifies regions of TspanC8s and ADAM10 for potential interaction-disrupting therapeutic targeting.

## Introduction

A disintegrin and metalloproteases (ADAMs)^2^ are one of the major classes of proteases that regulate transmembrane protein function, turnover and signaling (1, 2). ADAM10, and its most closely related family member ADAM17/TACE, are transmembrane zinc-dependent metalloproteases that contain an extracellular pro-domain, metalloprotease, disintegrin, and cysteine-rich and stalk domain, followed by a transmembrane region and C-terminal cytoplasmic domain. ADAM10 is ubiquitously expressed and has over 40 transmembrane protein substrates, which it cleaves within the extracellular region to release this region from the remaining transmembrane fragment. Important substrates for ADAM10 are the Notch cell fate regulators, as demonstrated by the embryonic lethality of ADAM10^−/−^ mice at e9.5, which phenocopies the Notch1^−/−^ phenotype (3). Other substrates include the amyloid precursor protein (APP), the IgE receptor CD23, EGF receptor ligands betacellulin and EGF, the platelet-activating collagen receptor GPVI, cadherins, and transmembrane chemokines (1, 2, 4, 5). As a result ADAM10 has been implicated as a potential target of modulation in diseases ranging from Alzheimer disease to heart disease and thrombosis to inflammation and cancer (1, 6, 7). Yet the regulation of ADAM10 itself and protein interactants that control ADAM10 activation and localization are only beginning to be characterized.

Three independent groups recently identified the TspanC8 subfamily of tetraspanin proteins as regulators of ADAM10 trafficking and maturation in multiple cell types and species (8–10). Tetraspanins are an evolutionarily conserved family of proteins, with 33 members in mammals, which contain four transmembrane spanning regions with two extracellular loops, one intracellular loop and intracellular N- and C-terminal tails. Tetraspanins interact with specific partner proteins and can form tetraspanin-enriched microdomains via tetraspanin-tetraspanin interactions. Tetraspanins regulate important aspects of partner protein function, in particular intracellular trafficking and lateral mobility and clustering at the plasma membrane (11, 12). The TspanC8 subgroup of tetraspanins consists of Tspan5, 10, 14, 15, 17, and 33 (8, 10). The TspanC8s promote ADAM10 maturation, which is the process by which the prodomain is cleaved by proprotein convertases during biosynthesis, and are required for ADAM10 exit from the endoplasmic reticulum (ER) and trafficking to the cell surface (8–10). There is evidence that different TspanC8s might promote ADAM10 shedding of specific substrates, since Tspan5, Tspan10, and Tspan14 are regulators of ADAM10-dependent Notch signaling, but Tspan15 is not (10, 13). This theory is supported by distinct TspanC8 subcellular localizations (10). This suggests that future therapeutic targeting to disrupt specific TspanC8-ADAM10 complexes might allow substrate- or cell type-specific ADAM10 targeting, while minimizing the toxic side effects that would result from global ADAM10 inhibition. However, the interacting regions of the TspanC8s and ADAM10 are not known, therefore such an approach cannot yet be undertaken.

The major aim of this study was to identify the regions of ADAM10 and TspanC8 proteins that are required to mediate their interaction. We identify these as the extracellular region of ADAM10 encompassing the cysteine-rich and stalk regions, and the large extracellular loops (LELs) of the TspanC8s. However, we present evidence that different TspanC8s interact with ADAM10 by distinct mechanisms. Moreover, we show that different TspanC8s can differentially affect cleavage of ADAM10 substrates.

## Experimental Procedures

### 

#### 

##### Antibodies

For Western blotting immunoprecipitation and immunofluorescence microscopy, primary antibodies were mouse anti-FLAG (M2) and rabbit anti-FLAG (Sigma), rabbit anti-HA (Cell Signaling Technologies (CST)), mouse anti-Myc (9B11) and rabbit anti-Myc (CST), mouse anti-human ADAM10, and goat anti-mouse ADAM10 (R&D Systems), mouse anti-CD9 (C9-BB) (14), mouse anti-human N-cadherin (BD Biosciences), rabbit anti-GFP (ab290), and mouse anti-human calnexin (AF18) (Abcam). The new goat anti-Tspan14 polyclonal was generated by Everest Biotech against a C-terminal cytoplasmic region of Tspan14 (SDIEAVKAGHH) that is identical between human and mouse.

##### Expression Constructs

N-terminal FLAG-tagged tetraspanin constructs were produced using the pEF6/Myc-His vector (Invitrogen) with an N-terminal FLAG tag (15), and cDNAs were cloned with stop codons to prevent C-terminal Myc-His tagging as described previously (8). The human FLAG-tagged Tspan14-CD9 chimera series of constructs were made by a two-step PCR method using overlapping PCR products as the second PCR template (16). For Tspan14, the LEL was amino acids 114–232 and the variable (Var) region 153–221, and for CD9, the LEL region was 112–192 and the Var region 152–181. The C-terminal HA-tagged mouse ADAM10 and ADAM17 in pcDNA3.1 (Invitrogen) have been described previously (17). Further chimeras of these constructs were made using the two-step PCR method described above. The ADAM10 disintegrin region consisted of amino acids 458–552, the cysteine-rich region 553–647, and the stalk region 648–673. ADAM17 disintegrin region was defined as amino acids 475–563, the cysteine-rich region 564–642, and the stalk region 643–671. The C-terminal Myc-tagged human ADAM10 in pRK5M was from Addgene (18). The truncated human ADAM10 constructs were generated by PCR and cloned into the pDisplay vector, which includes HA- and Myc-tag epitopes (Invitrogen). The human FcRγ and C-terminal GFP-tagged human GPVI constructs were as described (19).

##### Cell Culture and Transfections

The human embryonic kidney (HEK)-293T (HEK-293 cells expressing the large T-antigen of simian virus 40) and human HeLa epithelial cell lines were cultured in complete DMEM (cDMEM) medium (Sigma) that contains 10% fetal calf serum (Gibco), 4 mm l-glutamine, 100 units/ml penicillin, and 100 μg/ml streptomycin (PAA). Transient transfections in HEK-293T cells were carried out using polyethylenimine (Sigma) as described (20, 21). For N-cadherin and GPVI shedding experiments, 10 μm DAPT γ-secretase inhibitor and 10 μm GI254023X ADAM10 inhibitor (Sigma) were added 3 h post-transfection. For HeLa cell transfections, 2 μg of plasmid DNA was incubated in 250 μl of OptiMEM (Gibco) while 10 μl of Lipofectamine 2000 (Invitrogen) was incubated in 250 μl OptiMEM for 7 min before mixing and incubating for 25 min. The Lipofectamine-DNA mix was added to 4 × 10^5^ HeLa cells in 1.5 ml of cDMEM for 3 h before replacing the Lipofectamine-DNA mix for cDMEM. Human umbilical vein endothelial cells (HUVECs) were isolated and cultured as described previously (22), using umbilical cords with consent from the Birmingham Women's Health Care NHS Trust and approved by the Ethics Committee at the University of Birmingham.

##### Platelet Preparation

Human and mouse washed platelets were isolated from whole blood as previously described (23, 24). Consent for human blood was obtained from each donor, and platelet preparation was carried out with ethical approval.

##### Western Blotting and Co-immunoprecipitation

Experiments using primary cells were conducted using the following numbers of cells: 4 × 10^8^ human platelets per immunoprecipitation and 1 × 10^7^ per lane of whole cell lysate; 1.6 × 10^8^ mouse platelets per immunoprecipitation and 4 × 10^6^ per whole cell lysate; and 2.2 × 10^6^ HUVECs per immunoprecipitation and 5.5 × 10^4^ per whole cell lysate. Whole cell protein lysates and co-immunoprecipitation experiments were performed as previously described (8). Briefly, cells were lysed in 1% digitonin lysis buffer (10 mm Tris, pH 7.4, 150 mm NaCl, 0.02% NaN_3_). Proteins were immunoprecipitated with primary antibody (as indicated in text) bound to protein G-Sepharose beads for 90 min and washed in 0.1% digitonin lysis buffer. Standard protocols were used for Western blotting and SDS-PAGE. Primary antibodies were used as indicated in the text with corresponding horseradish peroxide (Pierce) or IRDye® 680RD or 800CW (LI-COR Biosciences)-conjugated secondary antibodies. Membranes were visualized using Pierce ECL Western blot substrate (Thermo Scientific) and exposure to film or using an Odyssey Infrared Imager (LI-COR Biosciences). All quantitation was performed using an Odyssey Infrared Imager (LI-COR Biosciences); background signal was removed, and individual band intensities were compared.

##### Flow Cytometry

For staining of ADAM10, transfected HeLa cells were scraped off the plate, and 5 × 10^5^ cells were stained with 10 μg/ml mouse anti-human ADAM10-APC or isotype control mouse IgG2b-APC (R&D Systems), and data were collected using CellQuest and a FACSCalibur (BD Biosciences). The geometric mean fluorescence intensity of isotype control staining was subtracted from the human ADAM10 staining to calculate ADAM10 expression.

##### Immunofluorescence Microscopy

Transfected HeLa cells were fixed, washed and blocked as described (22), prior to staining with primary antibodies. Subsequent staining was with wheat germ agglutinin (WGA)-FITC (Sigma) and/or secondary antibodies conjugated to Alexa488, Alexa568, or Alexa647 (Life Technologies). Images were captured on a Zeiss LSM 710 confocal microscope using a 40× objective.

##### Cell Surface Biotinylation

HEK-293T cells transfected with HA-tagged ADAM10 constructs were cell surface biotinylated as previously described (8). Cells were lysed in 1% Triton X-100 lysis buffer (10 mm Tris, pH 7.4, 150 mm NaCl, 1 mm EDTA, and 0.02% NaN_3_) containing protease inhibitors (Sigma), and anti-HA immunoprecipitates were analyzed by IRDye® 800CW-conjugated neutravidin (LI-COR Biosciences) Western blotting.

##### Statistics and Data Analysis

Relative or percentage data were log transformed and analyzed using a one-way ANOVA with a Dunnett's multiple comparison test using GraphPad Prism software. For comparison of different TspanC8 interactions with ADAM10 comprising the disintegrin, cysteine-rich domain, and stalk (DCS), ADAM10CS and ADAM10S (Fig. 10*A*), the Tspan14 co-immunoprecipitation with ADAM10DCS was first arbitrarily set to 100, and relative Tspan14 co-immunoprecipitations with ADAM10CS and ADAM10S were calculated, based on data from Fig. 8. Secondly, co-immunoprecipitations of the other TspanC8s with the three ADAM10 constructs were calculated relative to Tspan14, using data from Fig. 9.

## Results

### 

#### 

##### Generation of a New Tspan14 Antibody to Show That Tspan14 Interacts with ADAM10 in Primary Endothelial Cells and Platelets

**FIGURE 1.**
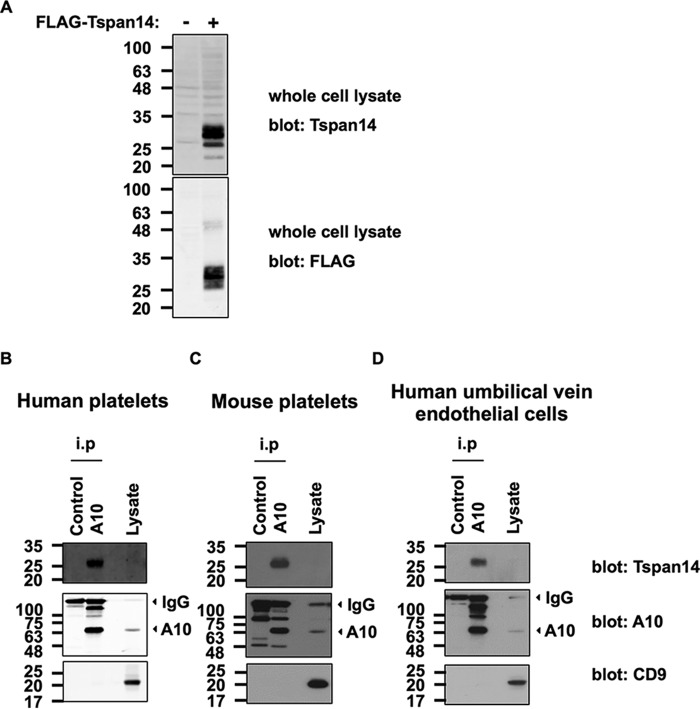
**Endogenous ADAM10 and Tspan14 interact in platelets and primary endothelial cells.** *A*, HEK-293T cells were mock transfected (−) or transfected with a FLAG-tagged human Tspan14 expression construct (+). The cells were lysed in 1% Triton X-100 lysis buffer and subjected to anti-Tspan14 (*top panel*) and anti-FLAG (*lower panel*) Western blotting. The Tspan14 antibody was raised in goat against a C-terminal cytoplasmic peptide, in collaboration with Everest Biotech. *B*, washed human platelets; *C*, washed mouse platelets and *D*, human umbilical vein endothelial cells were lysed in 1% digitonin lysis buffer, and proteins were immunoprecipitated with an antibody against ADAM10 or an isotype-matched control. Precipitates were then run on non-reducing gels, Western blotted, and probed with Tspan14 (*top panels*), ADAM10 (*middle panels*), and CD9 (*lower panels*) antibodies. *Arrows* indicate the positions of the predominant mature form of ADAM10 (A10) and the signal from the immunoprecipitating antibodies (IgG).

Previous data from our group and other groups demonstrated that the TspanC8 subfamily of tetraspanins interact with ADAM10 and are important for the maturation and cell surface expression of ADAM10 (8–10). However, these studies used co-immunoprecipitation of epitope-tagged proteins that were overexpressed in cell lines. To confirm that a TspanC8 can interact with ADAM10 at endogenous expression levels, we generated a polyclonal antibody to the C-terminal cytoplasmic tail of human Tspan14. Tspan14 was chosen as a model TspanC8 because we had previously shown this tetraspanin to regulate ADAM10 in primary endothelial cells (8). The antibody was first validated on FLAG-tagged human Tspan14 expressed in HEK-293T cells. Western blotting of whole cell lysates with the anti-Tspan14 antibody detected bands at 25–30 kDa for the FLAG-Tspan14 but not control transfections (Fig. 1*A*, *upper panel*). This correlated with bands detected by the anti-FLAG antibody (Fig. 1*A*, *lower panel*), confirming that the antibody detects Tspan14.

To test whether Tspan14 interacts with ADAM10 in primary cells, platelets and endothelial cells were chosen because Tspan14 is expressed in these cell types (8, 25, 26). Human platelets, mouse platelets, and human umbilical vein endothelial cells (HUVECs) were lysed in 1% digitonin lysis buffer, which we used previously to demonstrate ADAM10-TspanC8 interactions in transfected cells (8). ADAM10 or isotype control immunoprecipitates and cell lysate were then Western blotted with antibodies against ADAM10, Tspan14 or CD9, the latter as a non-TspanC8 control tetraspanin (Fig. 1, *B–D*). For each cell type, the Tspan14 antibody detected bands at 25–30 kDa from the ADAM10 immunoprecipitate, but this was absent from the control immunoprecipitate (Fig. 1, *B–D*, *top panels*). The 25–30 kDa size range is likely due to differential glycosylation of the single *N*-linked glycosylation site on Tspan14, as we have shown for another tetraspanin (15). Tspan14 was not detected in whole cell lysates, possibly because Tspan14 is expressed at relatively low levels. ADAM10 expression was confirmed in each ADAM10 immunoprecipitate (Fig. 1, *B–D*, *middle panels*). CD9 was undetectable in ADAM10 immunoprecipitates but was clearly observed in whole cell lysates (Fig. 1, *B–D*, *lower panels*), confirming the specificity of the ADAM10-Tspan14 interaction. These data are the first to show an endogenous ADAM10 interaction with a TspanC8 tetraspanin using specific antibodies.

##### The Large Extracellular Loop (LEL) of Tspan14 Is Required to Interact with ADAM10

**FIGURE 2.**
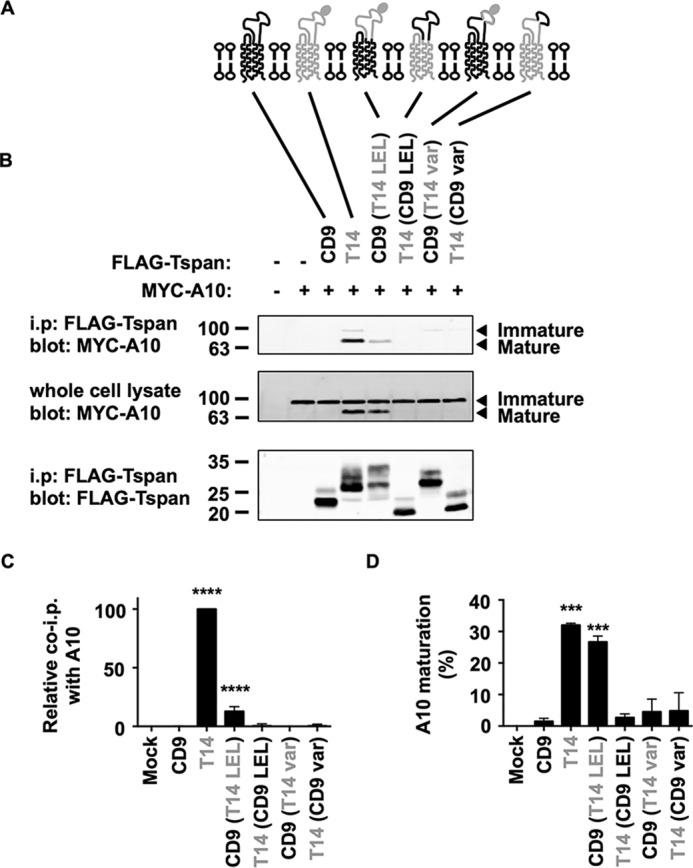
**The large extracellular loop (LEL) of Tspan14 is the region that interacts with ADAM10 and is required for ADAM10 maturation.** *A*, schematic of Tspan14 and CD9 chimeras. The large extracellular loop (LEL) and variable (var) region of CD9 (*black*) and Tspan14 (*gray*) were interchanged; the *N*-linked glycosylation site of Tspan14 is indicated by a *filled oval. B*, HEK-293T cells were mock transfected (−) or transfected with expression constructs containing the FLAG-tagged human tetraspanin chimeras with Myc-tagged human ADAM10 (+). Cell lysates were produced using 1% digitonin lysis buffer and immunoprecipitated with an anti-FLAG antibody. Immunoprecipitated proteins were blotted with anti-Myc tag antibody (*top panel*) or anti-FLAG antibody (*lower panel*). Whole cell lysates were probed with the anti-Myc tag antibody (*middle panel*). Data are representative of three independent experiments. *C*, quantitation of immunoprecipitated ADAM10. Data in *panel B* (*upper panel*) were quantitated using the Odyssey Infrared Imaging System (LI-COR), and the amount of ADAM10 immunoprecipitated was shown relative to immunoprecipitated Tspan14, which was arbitrarily set at 100. Data were normalized by log transformation and statistically analyzed using a one-way ANOVA with a Dunnett's multiple comparison test compared with the mock (****, *p* < 0.0001). Error bars represent standard error of the mean from three experiments. *D*, data in panel B (*middle panel*) were quantitated, the percentage of mature ADAM10 calculated, and the data log transformed and statistically analyzed as described for *panel C* (***, *p* < 0.001).

To determine the region of Tspan14 required for interaction with ADAM10, four FLAG-tagged human Tspan14 and CD9 chimeras were made (Fig. 2*A*); CD9 was chosen as a representative non-TspanC8 tetraspanin. These chimeras involved exchange of the entire LELs, or exchange of the so-called variable regions of the LEL that are relatively divergent in sequence, since these have been implicated in mediating interactions between other tetraspanins and their partners (27). These FLAG-tagged chimeric tetraspanins were then co-expressed with Myc-tagged human ADAM10 in HEK-293T cells. Cells were lysed using 1% digitonin lysis buffer, tetraspanins were immunoprecipitated with an anti-FLAG antibody, and immunoprecipitates were separated by SDS-PAGE and probed with anti-Myc and anti-FLAG antibodies to detect ADAM10 and tetraspanins, respectively. The only chimera that co-immunoprecipitated with ADAM10 comprised of CD9 with the Tspan14 LEL (Fig. 2*B*, *upper panel*), and this interaction was significant but with a substantially lower efficiency than wild-type Tspan14 (Fig. 2*C*). In addition, this was the only chimera that promoted ADAM10 maturation, as detected by anti-Myc blotting of whole cell lysates (Fig. 2, *B*, *middle panel*, and *D*). Similar levels of immunoprecipitation were achieved for each of the chimeras, as detected by anti-FLAG blotting of the immunoprecipitates (Fig. 2*B*, *lower panel*). These data suggest that, in the context of chimeric tetraspanins, the LEL of Tspan14 is necessary and sufficient to interact with ADAM10 and promote its maturation, whereas the variable region of the LEL is also necessary but not sufficient.

**FIGURE 3.**
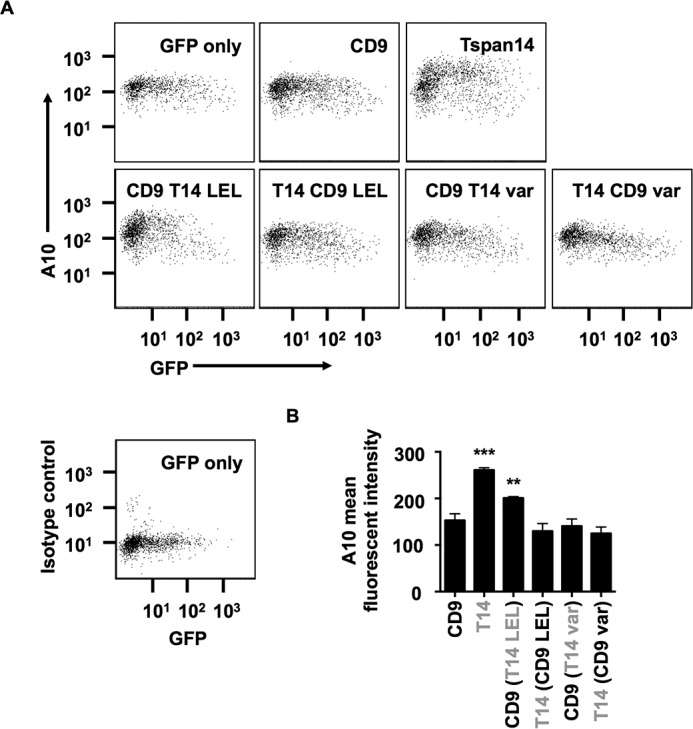
**The large extracellular loop (LEL) of Tspan14 is critical for its ability to increase ADAM10 cell surface accumulation.** *A*, HeLa cells were transfected with the indicated Tspan14-CD9 chimeras (see Fig. 2*A*) and GFP to identify transfected cells. Cells were stained with an APC-conjugated ADAM10 antibody and analyzed by flow cytometry. *Dot plots* are representative of three independent experiments. The *bottom left panel* shows isotope control staining. *B*, average geometric mean fluorescent intensities for ADAM10 staining, gated on live and GFP-positive cells, were compared statistically using a one-way ANOVA with a Dunnett's multiple comparison test, compared with the CD9 control (***, *p* < 0.001; **, *p* < 0.01). Error bars represent standard error of the mean from three experiments.

**FIGURE 4.**
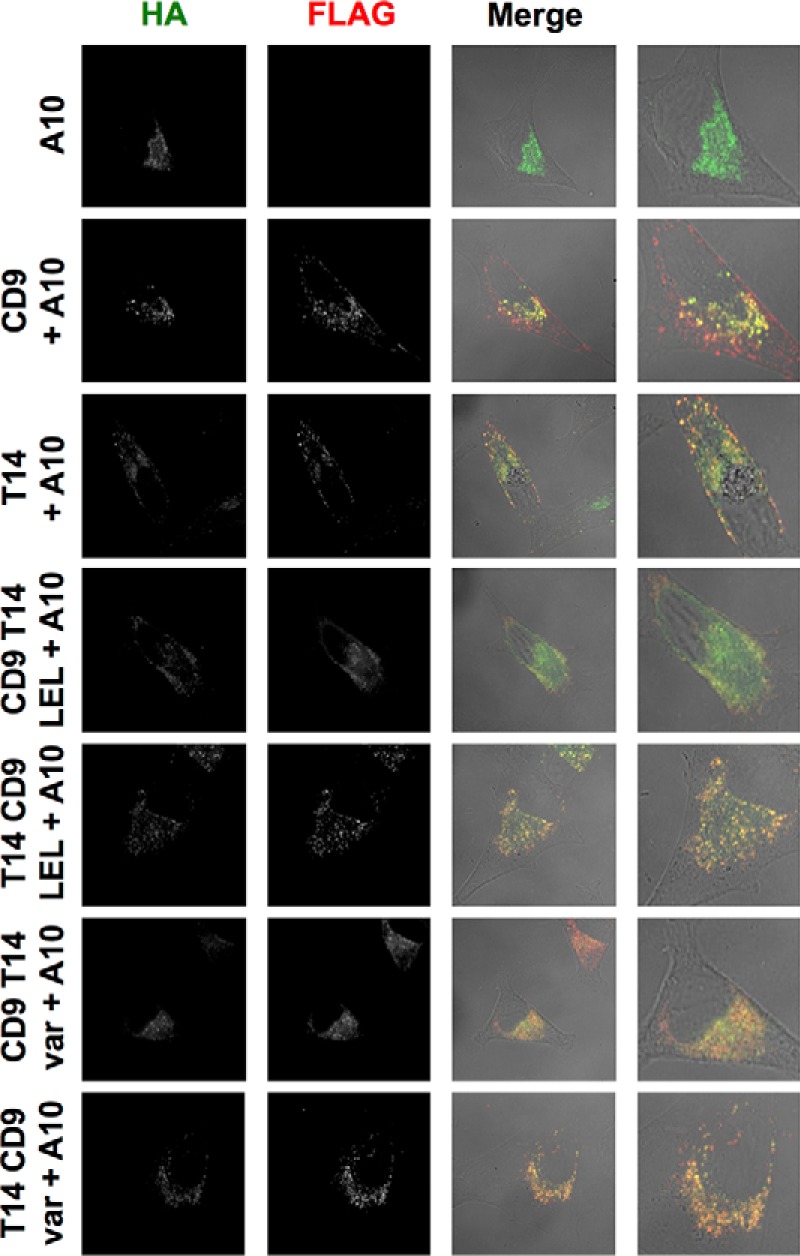
**All Tspan14-CD9 chimeras partially co-localize with ADAM10 and so have access to the metalloprotease.** HeLa cells were transfected with the indicated Tspan14-CD9 chimeras (see Fig. 2*A*) and HA-tagged mouse ADAM10. Cells were fixed and stained with an anti-HA antibody (*green*) and an anti-FLAG antibody (*red*). Confocal microscopy images are representative of three independent experiments and at least 15 fields of view.

Dornier *et al.* demonstrated that Tspan14 over-expression is able to increase the surface expression of ADAM10 in the HeLa cell line (10). To investigate whether these CD9-Tspan14 chimeras can increase cell surface expression of endogenous ADAM10 in HeLa cells, each chimera was co-expressed with GFP, to label the transfected cells, and flow cytometry was used to determine surface expression of ADAM10. Consistent with the interaction and maturation data in Fig. 2, only the CD9-Tspan14 LEL chimera and wild-type Tspan14 significantly elevated ADAM10 surface expression (Fig. 3, *A* and *B*). To confirm that each of these chimeras had access to ADAM10 and were not simply localized to a different subcellular compartment, co-immunofluorescence confocal microscopy was performed in transfected HeLa cells. Some co-localization between each chimera and ADAM10 was observed (Fig. 4), even for those which did not co-immunoprecipitate with ADAM10 or promote its maturation or cell surface expression. Together these data provide evidence that the LEL of Tspan14 mediates the interaction with ADAM10 to promote its maturation and trafficking to the cell surface.

##### The Combined Disintegrin, Cysteine-rich, and Stalk Regions of ADAM10 Can Mediate the Interaction with TspanC8s

**FIGURE 5.**
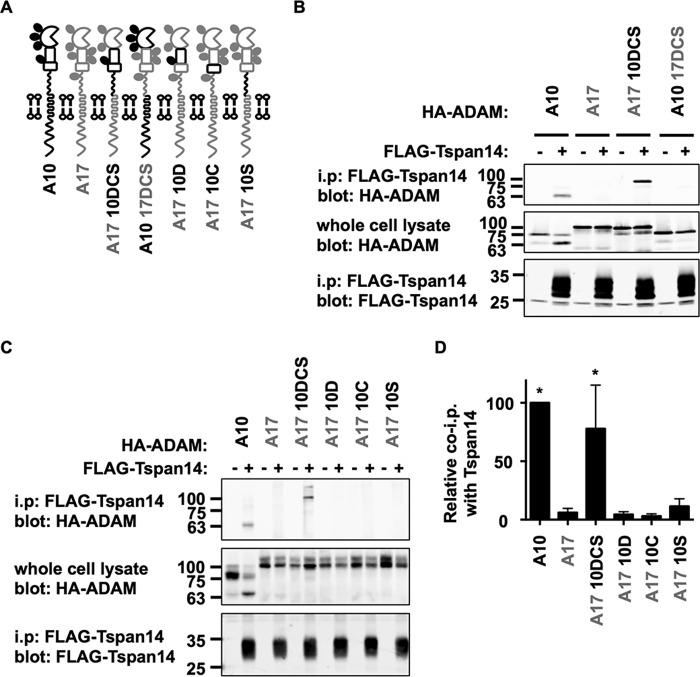
**The region of ADAM10 comprising the disintegrin domain (*D*), the cysteine-rich (*C*), and stalk (*S*) regions mediates the interaction with Tspan14.** *A*, schematic of ADAM10 and ADAM17 chimeras. The extracellular disintegrin (*D*), cysteine-rich (*C*), and stalk (*S*) regions of ADAM10 (*black*) and ADAM17 (*gray*) were interchanged together (*DCS*) or individually. *B*, HEK-293T cells were mock transfected (−) or transfected with FLAG-tagged mouse Tspan14 (+) in addition to either HA-tagged mouse ADAM10, ADAM17, ADAM17 10DCS, or ADAM10 17DCS. Cells were lysed in 1% digitonin lysis buffer and immunoprecipitated with an anti-FLAG antibody. Immunoprecipitated proteins were blotted with anti-HA tag antibody (*top panel*) or anti-FLAG antibody (*lower panel*). Whole cell lysates were probed with the anti-HA tag antibody (*middle panel*). The blots are representative of three independent experiments. *C*, HEK-293T cells were co-transfected with (+) or without (−) FLAG-tagged mouse Tspan14 and either HA-mouse ADAM10, ADAM17, ADAM17 10DCS, ADAM17 10D, ADAM17 10C, or ADAM17 10S. Cells were treated as in *B. D*, data from *panels B* and *C* were quantitated and presented as the relative amount of each ADAM10/17 construct immunoprecipitated with Tspan14, having arbitrarily set wild-type ADAM10 to 100. Data were normalized by log transformation and statistically analyzed using a one-way ANOVA with a Dunnett's multiple comparison test, compared with the ADAM17 control (*, *p* < 0.05). Error bars represent standard errors of the mean from 3–6 experiments.

Having determined that the LEL region of Tspan14 interacted with ADAM10, we focused on the membrane-proximal extracellular domains of ADAM10, namely the disintegrin (D), cysteine-rich (C), and stalk (S) domains, as the regions potentially involved in Tspan14 binding. Again a chimeric approach was employed, using the ADAM10-related ADAM17 (Fig. 5*A*). HA-tagged mouse ADAM10-ADAM17 chimeras were co-expressed in HEK-293T cells with or without FLAG tagged mouse Tspan14, the cells lysed in 1% digitonin and subjected to anti-FLAG immunoprecipitation. The chimera comprising ADAM17 with the ADAM10 DCS region co-immunoprecipitated with Tspan14, but the chimera of ADAM10 with the ADAM17 DCS region did not (Fig. 5*B*). As controls, ADAM10 co-immunoprecipitated with Tspan14 but ADAM17 did not (Fig. 5*B*). This suggests that the region of ADAM10 encompassing the disintegrin, cysteine-rich, and stalk region is necessary and sufficient to interact with Tspan14. To determine whether any of these regions alone were sufficient for the interaction, further chimeras were generated of ADAM17 containing each of the three individual ADAM10 domains. However, none of these individual ADAM10 extracellular domains enabled interaction with Tspan14 in the ADAM17 backbone (Fig. 5, *C* and *D*). It is possible that some of these chimeras might not be folded correctly. Nevertheless, the data suggest that the entire disintegrin, cysteine-rich and stalk region may be important to mediate the ADAM10-Tspan14 interaction.

**FIGURE 6.**
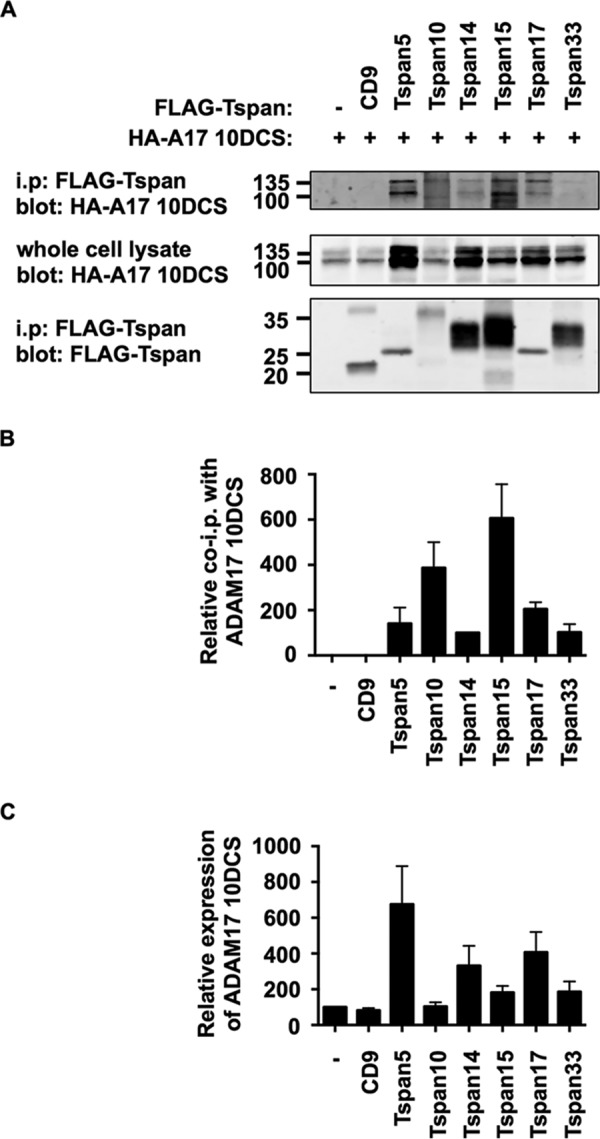
**All TspanC8s interact with the region of ADAM10 comprising the disintegrin (*D*), cysteine-rich domain (*C*), and stalk (*S*).** *A*, HEK-293T cells were transfected with expression constructs for the HA-tagged mouse ADAM17 10DCS chimera and FLAG-tagged mouse TspanC8s, CD9 or negative control (−). Lysates were extracted in 1% digitonin lysis buffer and proteins immunoprecipitated with an anti-FLAG antibody. Immunoprecipitates were blotted with anti-HA tag antibody (*top panel*) or anti-FLAG antibody (*lower panel*). Whole cell lysates were probed with the anti-HA tag antibody (*middle panel*). *B*, data in panel A (*upper panel*) were quantitated, and the amount of ADAM17 10DCS immunoprecipitated was normalized for the amount in the whole cell lysate. Data are shown relative to immunoprecipitated Tspan14, which was arbitrarily set at 100. Data were normalized by log transformation and statistically analyzed using a one-way ANOVA with a Dunnett's multiple comparison test compared with the mock. All TspanC8s bound significantly to ADAM17 10DCS (*p* < 0.0001). Error bars represent standard error of the mean from three experiments. *C*, ADAM17 10DCS whole cell lysate data in *panel A* were quantitated, and the amount of ADAM17 10DCS expressed was normalized to the expression in the first lane, which was arbitrarily set at 100. Error bars represent standard error of the mean from three experiments.

As all TspanC8s interact with ADAM10, we sought to examine whether each TspanC8 behaved similarly to Tspan14 by interacting with the region of ADAM10 comprising the disintegrin, cysteine-rich domain and stalk. Each of the FLAG-tagged mouse TspanC8 family members, or CD9 as a control, was expressed in HEK-293T cells with the ADAM17 10DCS chimera. Anti-FLAG immunoprecipitations were performed as described above. All six of the TspanC8 family members significantly interacted with the ADAM17 10DCS chimera, but there were differences in the efficiency of the interactions (Fig. 6, *A* and *B*). In particular, the interactions with Tspan10 and 15 were substantially stronger than for other TspanC8s (Fig. 6*B*). This is in contrast to the similar levels of interaction previously observed for each TspanC8 with wild-type ADAM10 (8). Tspan5, and to a lesser extent Tspan14 and 17, consistently resulted in higher expression levels of the ADAM17 10DCS chimera in whole cell lysates (Fig. 6, *A* and *C*). It is possible that these TspanC8s can promote the stability of this chimera. Together these data suggest that different TspanC8s might bind to ADAM10 via subtly different mechanisms, and that this can be revealed by co-immunoprecipitation with the ADAM17 10 DCS chimera.

**FIGURE 7.**
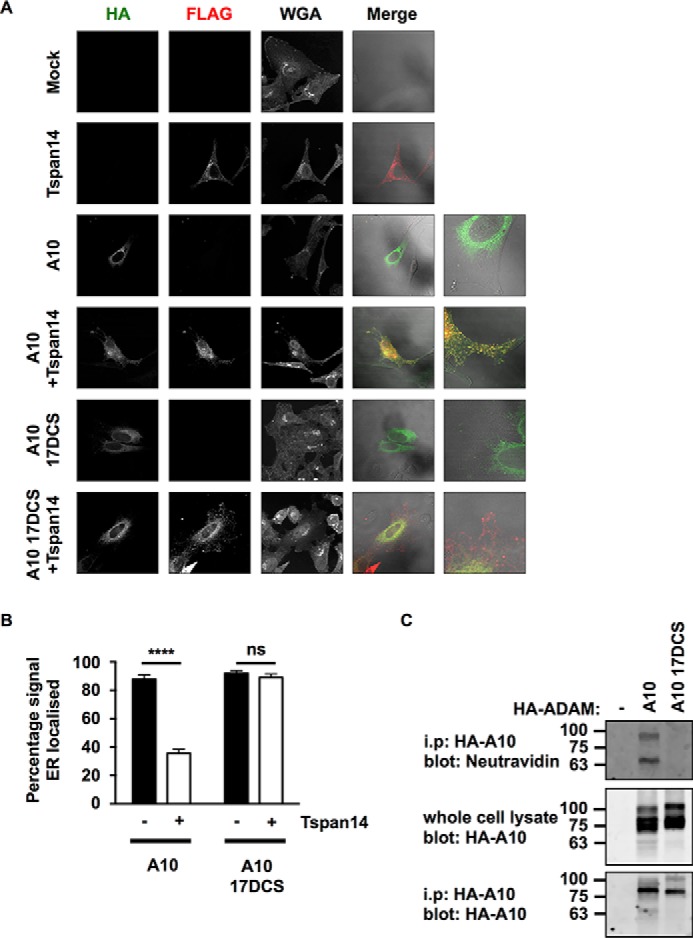
**The disintegrin (*D*), cysteine-rich (*C*), and stalk (*S*) regions of ADAM10 are essential for Tspan14-mediated exit from the ER.** *A*, HeLa cells were transfected with combinations of FLAG-tagged Tspan14 and HA-tagged mouse ADAM10 wild-type or ADAM10 17DCS. Cells were fixed and stained with an anti-HA antibody (*green*), an anti-FLAG antibody (*red*) and WGA to visualize the plasma membrane and internal cellular structures by confocal microscopy. *B*, HeLa cells were transfected and stained as in *panel A* except an anti-calnexin antibody was used instead of WGA to define the limits of the ER (images not shown). The HA signal was quantitated across the whole cell and within the mask of the calnexin staining, and presented as a percentage of HA-ADAM10 or HA-ADAM10 17DCS signal localized in the ER. Data are representative of three independent experiments and at least 15 fields of view. A two-way ANOVA statistical analysis was performed with a Bonferroni's multiple comparisons test (*ns*, non-significant, ****, *p* < 0.0001). *C*, HEK-293T cells were mock transfected (−), or transfected with HA-tagged mouse ADAM10 wild-type or ADAM10 17DCS. Cells were surface biotinylated, lysed, and immunoprecipitated with an anti-HA antibody. Immunoprecipitates were stained with neutravidin (*top panel*) or an anti-HA antibody (*bottom panel*). Whole cell lysates were stained with an anti-HA antibody (*middle panel*).

To investigate how an inability to interact with TspanC8s impacts ADAM10, the HA-tagged ADAM10 17DCS chimera was transfected into HeLa cells in the presence or absence of FLAG-tagged Tspan14. Immunofluorescence confocal microscopy showed a perinuclear localization for the ADAM10 17DCS chimera, which did not appear to colocalize with Tspan14 (Fig. 7*A*). In contrast, wild-type ADAM10 was not restricted to the perinuclear region when co-expressed with Tspan14 and strongly co-localized with Tspan14 (Fig. 7*A*). Co-staining with an anti-calnexin antibody, to label the ER (images not shown), revealed that the ADAM10 17DCS chimera was largely ER-restricted in the presence of Tspan14, unlike wild-type ADAM10 (Fig. 7*B*). To confirm that interaction with TspanC8s is necessary for ADAM10 cell surface expression, a cell surface biotinylation approach was used with transfected HEK-293T cells. No biotinylation of the ADAM10 17DCS chimera was detected, in contrast to wild-type ADAM10 (Fig. 7*C*). These data show that interaction with a TspanC8 is required for ADAM10 to exit the ER and thus for normal ADAM10 function, consistent with previous studies showing that TspanC8s are required for ADAM10 ER exit (9, 10).

##### The Combined Cysteine-rich and Stalk Regions of ADAM10 Mediate the Interaction with Tspan14

**FIGURE 8.**
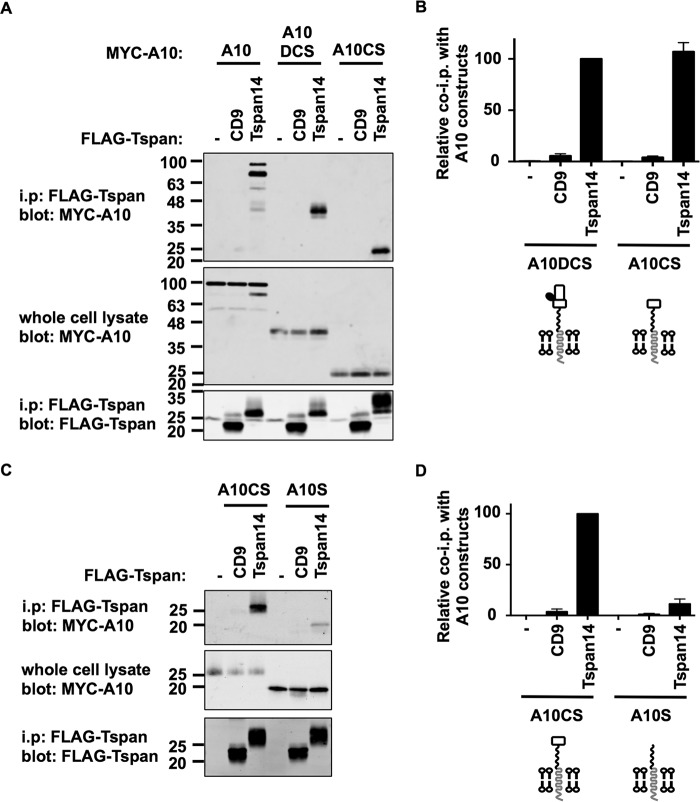
**The combined cysteine-rich (*C*) and stalk (*S*) region of ADAM10 without the disintegrin (*D*) is sufficient to interact with Tspan14.** *A*, HEK-293T cells were mock transfected (−) or transfected with FLAG-tagged human CD9 or Tspan14, with co-transfection of Myc-tagged human ADAM10, or pDisplay constructs containing ADAM10DCS or ADAM10CS, which also possessed Myc tags. Cells were lysed in 1% digitonin lysis buffer and immunoprecipitated with an anti-FLAG antibody. Immunoprecipitated proteins were blotted with anti-Myc tag antibody (*top panel*) or anti-FLAG antibody (*lower panel*). Whole cell lysates were probed with the anti-Myc tag antibody (*middle panel*). *B*, data in panel A (*upper panel*) were quantitated from three experiments. Data were log transformed and compared statistically with a one-way ANOVA with a Dunnett's multiple comparison test against the mock. Tspan14 bound significantly to ADAM10DCS (*p* < 0.0001) and ADAM10CS (*p* < 0.0001). A diagrammatic representation of the ADAM10 constructs is shown *below* the graph. *C*, HEK-293T cells were mock transfected (−) or transfected with FLAG-tagged human CD9 or Tspan14, with co-transfection of pDisplay ADAM10CS or ADAM10S. Cells were treated as in *panel A. D*, data in *panel C* were quantitated from three experiments. Data were log transformed and compared statistically with a one-way ANOVA with a Dunnett's multiple comparison test against the mock. Tspan14 bound significantly to ADAM10CS (*p* < 0.0001) and ADAM10S (*p* < 0.001).

To further isolate the region of ADAM10 with which Tspan14 interacts, truncations of the human ADAM10 DCS region were expressed using the pDisplay expression vector. This utilizes the murine Ig κ-chain leader sequence to display the intended protein at the cell surface with HA and Myc tags, fused to the transmembrane domain of platelet derived growth factor receptor. Using transfected HEK-293T cells and anti-FLAG tetraspanin immunoprecipitations as described previously, comparable levels of co-immunoprecipitation were observed for ADAM10 wild-type, DCS or CS truncation constructs with Tspan14 (Fig. 8, *A* and *B*). However, truncation down to just the ADAM10 stalk substantially reduced the ability of Tspan14 co-immunoprecipitate with ADAM10 (Fig. 8, *C* and *D*). As controls in each of these experiments, no ADAM10 was detected in immunoprecipitations from CD9 or mock co-transfections (Fig. 8, *A–D*). The increase in Tspan14 molecular weight when co-expressed with ADAM10 CS or S truncation constructs was consistent (Fig. 8, *A* and *C* and data not shown), and is likely due to differential glycosylation of its single *N*-linked site. Together these data suggest that the minimal extracellular regions of ADAM10 required for substantial binding to Tspan14 are the cysteine-rich domain and stalk.

##### The TspanC8 Subfamily Proteins Bind Differentially to the Disintegrin, Cysteine-rich, and Stalk Regions of ADAM10

**FIGURE 9.**
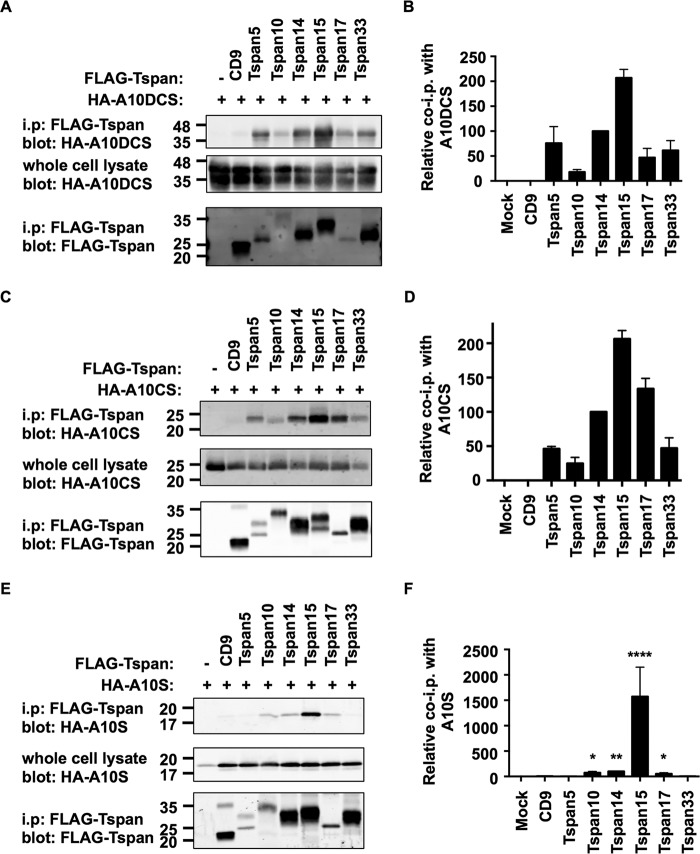
**The TspanC8s bind differentially to the disintegrin (*D*), cysteine-rich (*C*), and stalk (*S*) regions of ADAM10.** *A*, HEK-293T cells were mock transfected (−) or transfected with FLAG-tagged mouse TspanC8s or CD9, and co-transfected with the pDisplay vector containing HA-tagged human ADAM10DCS. Cell lysates were produced in 1% digitonin lysis buffer and immunoprecipitated with an anti-FLAG antibody. Immunoprecipitated proteins were blotted with anti-HA tag antibody (*top panel*) or anti-FLAG antibody (*lower panel*). Whole cell lysates were probed with the anti-Myc tag antibody (*middle panel*). *B*, data from panel A (*upper panel*) were quantitated and presented as the amount of immunoprecipitated ADAM10DCS relative to the Tspan14 immunoprecipitation, which was arbitrarily set to 100. Data were normalized by log transformation and statistically analyzed using a one-way ANOVA with a Dunnett's multiple comparison test compared with the CD9 control. All TspanC8s bound significantly to ADAM10DCS (*p* < 0.001). Error bars represent the standard error of the mean from three experiments. *C* and *D*, these experiments were carried out as described for *panels A* and *B* except using HA-tagged human ADAM10CS. All TspanC8s bound significantly to ADAM10DCS (*p* < 0.0001). *E* and *F*, these experiments were carried out as for *panels A* and *B* except using HA-tagged human ADAM10S (****, *p* < 0.0001; **, *p* < 0.01; *, *p* < 0.05).

To determine whether, like Tspan14, each of the other TspanC8 subfamily members require the ADAM10 cysteine-rich and stalk regions for minimal binding, they were compared with Tspan14 for co-immunoprecipitation with each truncated ADAM10 construct. All TspanC8s co-immunoprecipitated with the ADAM10 DCS truncation (Fig. 9, *A* and *B*). Similarly, all TspanC8s interacted with the CS truncation of ADAM10 (Fig. 9, *C* and *D*). Finally, only Tspan15 interacted substantially with the S truncation representing just the stalk region of ADAM10 (Fig. 9, *E* and *F*). Tspan10, 14, and 17 each interacted weakly but significantly with the stalk region, while Tspan5 and 33 did not interact at all (Fig. 9, *E* and *F*).

**FIGURE 10.**
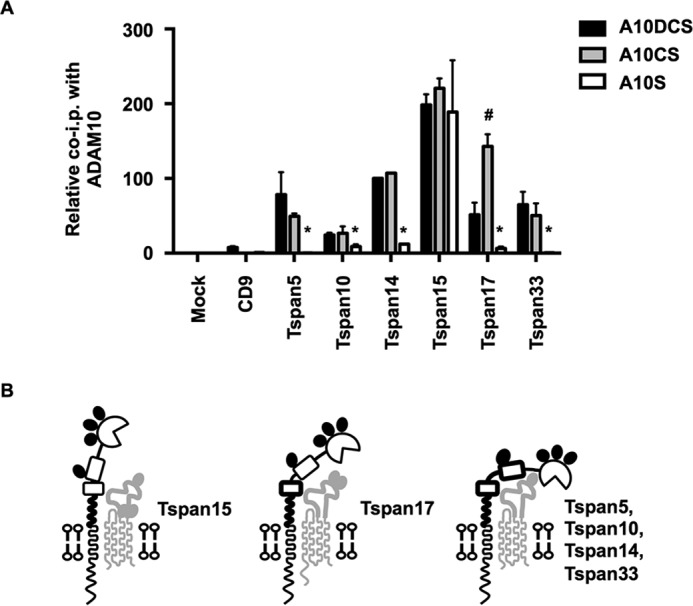
**Evidence that different TspanC8s interact with ADAM10 by distinct mechanisms.** *A*, comparison of TspanC8 co-immunoprecipitations with ADAM10 truncation constructs. Quantitation of the co-immunoprecipitations of ADAM10DCS, ADAM10CS, and ADAM10S with each tetraspanin from Fig. 9 were compared. Values were normalized using Tspan14 data from Fig. 8. All data were relative to the co-immunoprecipitation of ADAM10DCS with Tspan14, which was arbitrarily set to 100. Data were log transformed and statistical analysis was performed using a one-way ANOVA with a Dunnett's multiple comparison test comparing ADAM10CS (#, *p* < 0.01) or ADAM10S (*, *p* < 0.01) to the ADAM10DCS for each tetraspanin. Error bars represent the standard error of the mean from three experiments. *B*, schematic of the potential differential modes of interaction of the TspanC8s with ADAM10. *Bold regions* of ADAM10 represent those required for a strong interaction with the corresponding TspanC8. Note that Tspan15 has 3 *N*-linked glycosylation sites and Tspan17 has 2, whereas Tspan5, 10, 14, and 33 have 3, 0, 1, and 2, respectively; for the latter, Tspan14 is depicted as an example.

To enable a direct comparison of TspanC8 interactions with the different ADAM10 truncation mutants, the quantitated data in Figs. 8 and 9 were combined and adjusted to make all values relative (Fig. 10*A*). This analysis mitigated differences in expression between the TspanC8s by directly comparing each TspanC8 with itself, for the different ADAM10 truncations. Tspan15 bound equally to each of the ADAM10 truncations, indicating that the major contact site for Tspan15 is within the ADAM10 stalk region (Fig. 10*A*). For all other TspanC8s, loss of the cysteine-rich region significantly reduced the interaction with ADAM10 (Fig. 10*A*). Furthermore, the Tspan17 interaction with the stalk and cysteine-rich region was inhibited by the presence of the disintegrin domain (Fig. 10*A*). These findings, represented in diagrammatic form in Fig. 10*B*, suggest that the six TspanC8s have key differences in their mechanisms of interaction with the region of ADAM10 encompassing the disintegrin, cysteine-rich, and stalk regions.

##### Differential Effects of TspanC8s on ADAM10 Substrate Cleavage: Tspan15 Promotes Cleavage of N-cadherin and Tspan14 Reduces Cleavage of GPVI

**FIGURE 11.**
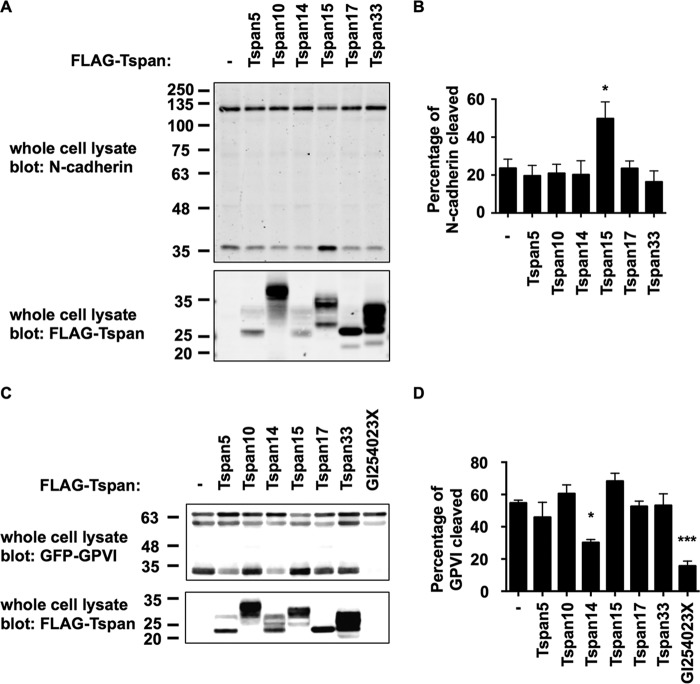
**Differential effects of TspanC8s on ADAM10 substrate cleavage: Tspan15 promotes cleavage of N-cadherin and Tspan14 reduces cleavage of GPVI.** *A*, HEK-293T cells were mock transfected (−) or transfected with FLAG-tagged mouse TspanC8s. The cells were lysed in 1% Triton X-100 lysis buffer and subjected to Western blotting with an antibody to the C-terminal cytoplasmic tail of N-cadherin (*upper panel*) or with an antibody to the FLAG epitope (*lower panel*). *B*, data from *A* (*upper panel*) were quantitated and the *lower, cleaved band* given as a percentage of the total (*upper and lower band* combined). Data were normalized by log transformation and statistically analyzed using a one-way ANOVA with a Dunnett's multiple comparison test compared with the mock control. Error bars represent the standard error of the mean from three experiments (*, *p* < 0.05). *C*, HEK-293T cells were co-transfected with GPVI and FcRγ and one of each of the FLAG-tagged mouse TspanC8s or without a tetraspanin (−) or with the addition of the ADAM10 inhibitor GI254023X at 10 μm. Cells were treated as in *panel A*, except lysates were subjected to an anti-GFP antibody (*upper panel*) instead of an anti-N-cadherin antibody. *D*, data from panel C (*upper panel*) were quantitated as described in *panel A* (***, *p* < 0.001).

To assess whether the TspanC8s also have differential effects on cleavage of an endogenous ADAM10 target, the adhesion molecule N-cadherin was selected due to its expression in HEK-293T cells and because it appears to be specifically cleaved by ADAM10 (28). Cleavage was detected using an antibody to the C-terminal cytoplasmic tail of N-cadherin, by Western blotting lysates of HEK-293T cells over-expressing one of each of the FLAG-tagged TspanC8s. Tspan15, but not the other TspanC8s, promoted a significant increase in the relative amount of the C-terminal fragment of N-cadherin *versus* full-length (Figs. 11*A*, *upper panel*, and 11*B*). This promotion of N-cadherin cleavage by Tspan15 was likely to be more substantial than indicated by the quantitation (Fig. 11*B*), because only ∼50% of cells were transfected in these experiments, as assessed by flow cytometry of co-transfected green fluorescent protein (data not shown). This finding was not a consequence of TspanC8 expression levels, because anti-FLAG Western blotting demonstrated that Tspan15 was not the most highly expressed TspanC8 (Fig. 11*A*, *lower panel*). These data suggest a specific role for Tspan15 in promoting ADAM10 cleavage of N-cadherin.

To determine whether Tspan15 or other TspanC8s might promote cleavage of an additional substrate, the platelet collagen receptor GPVI was selected as a known substrate of ADAM10 (4, 5). Since GPVI is not expressed by HEK-293T cells, they were co-transfected with constructs for GPVI with a cytoplasmic GFP tag, the GPVI-associated FcRγ chain and TspanC8s. Anti-GFP Western blotting showed that expression of Tspan14 significantly reduced GPVI cleavage, almost to the same extent as the ADAM10 inhibitor GI254023X (Fig. 11*C*). None of the other TspanC8s significantly altered GPVI cleavage (Fig. 11*D*). Interestingly, Tspan14 is relatively highly expressed in the megakaryocyte/platelet lineage (8), and so may protect GPVI from cleavage in this cell lineage. Together with our N-cadherin data and that previously reported for Notch (10), these findings suggest that ADAM10 substrate specificity may be dictated by the TspanC8 with which it is associated.

## Discussion

TspanC8 tetraspanins were previously shown to interact with ADAM10 in overexpression systems (8, 10). In this study, we generated an antibody to Tspan14 as a representative TspanC8, which we used to demonstrate that Tspan14 interacts with ADAM10 endogenously in human and mouse primary cells. To identify the ADAM10-interacting region of Tspan14, we concentrated on the LEL, since this region on other tetraspanins facilitates many characterized tetraspanin-partner protein interactions and is the most divergent region of tetraspanins, making this a likely partner protein binding surface (29–31). Using chimeras of Tspan14 and CD9, we demonstrated that the LEL of the tetraspanin mediates its interaction with ADAM10. The variable region of this LEL was also critical but not sufficient. The CD9-Tspan14 LEL chimera did not co-immunoprecipitate ADAM10 to the same level or increase cell surface expression as much as wild-type Tspan14. This difference may be due to insufficient stabilization of the LEL of Tspan14 by the small extracellular loop (SEL) of CD9 in the chimera. The SEL of Tspan14 is predicted to be just 19 amino acids compared with 24 amino acids for CD9, with no sequence homology between them, and it has been hypothesized that the SEL interacts with the hydrophobic interface of the LEL N-terminal linker (32). Nevertheless, our demonstration that the LEL of Tspan14 was important for its interaction with ADAM10 is analogous to similar data for other tetraspanin-partner protein interactions. The interaction of CD151 with α3β1 integrins has been extensively studied using a similar chimeric tetraspanin approach, demonstrating that the variable region of the LEL is required for α3β1 integrin binding, and antibodies that target the LEL also disrupt the interaction (33–35). Association of another tetraspanin, CD81, with its partner protein CD19 is also mediated by the LEL region (36). In addition, CD81 facilitates CD19 surface expression and exit from the endoplasmic reticulum (37), similar to the regulation of ADAM10 maturation and surface expression by the TspanC8s (8–10). However, for CD19 this also requires transmembrane domain 1 of CD81 (36). For EWI-2 binding to the related tetraspanins CD9 and CD81, the CD81 LEL and transmembrane domains 3 and 4 are required, but for CD9 the LEL and transmembrane domains 2 and 3 are required (38). A CD82-CD81 LEL chimera is not sufficient for binding to EWI-2 (38), yet the LEL of CD9 is able to facilitate binding to EWI-2 (39), although it is substantially reduced, similar to that observed for our CD9-Tspan14 LEL chimera binding to ADAM10. It is therefore possible that a chimera containing additional transmembrane regions of Tspan14 may increase binding to wild-type Tspan14 levels.

Despite over 40 known substrates for ADAM10, very few proteins have been shown to directly interact, biochemically, with ADAM10. The TspanC8s are the only proteins known to alter ADAM10 maturation and intracellular trafficking. Using chimeras of ADAM10 and ADAM17, we have discovered that the membrane-proximal regions of ADAM10, including the stalk, cysteine-rich and disintegrin regions, are required for TspanC8-ADAM10 interaction. To further isolate the interaction region, we used a series of pDisplay constructs with truncation of the extracellular region of ADAM10. Strikingly, the 26 amino acid stalk region of ADAM10 was sufficient for interaction with Tspan15, and this was not increased by inclusion of cysteine-rich and disintegrin domains. Tspan10, Tspan14, and Tspan17 each interacted relatively weakly to the stalk region, while Tspan5 and Tspan33 did not interact at all. Each of these five TspanC8s exhibited substantial interactions with the stalk plus cysteine-rich region of ADAM10. These interactions were not enhanced by the additional inclusion of the disintegrin domain and, for Tspan17, the interaction was partially impaired. These data suggest that different TspanC8s engage ADAM10 in subtly different ways, which may have implications for ADAM10 function. In particular, ADAM10 may have multiple conformations that are stabilized by different TspanC8s. Dornier *et al.* previously demonstrated that ADAM10-mediated activation of a Notch reporter is promoted by Tspan5 and Tspan14 expression, but not by Tspan15 (10). We have now demonstrated that Tspan15, but not the other TspanC8s, promotes ADAM10-mediated N-cadherin cleavage, and that Tspan14 reduces GPVI cleavage. We propose that different TspanC8s might direct substrate specificity by constraining ADAM10 into defined conformations (Fig. 10*B*), and that the distinct Tspan15-ADAM10 interaction mechanism may favor cleavage of certain substrates such as N-cadherin, but may prevent cleavage of others such as Notch. An alternative, and currently unexplored, possibility is that TspanC8s could regulate ADAM10 substrate selectivity by directly binding to the substrates.

We have previously shown Tspan14 to be the most highly expressed TspanC8 in mouse megakaryocytes at the mRNA level (8). In the present study, we have used our new Tspan14 antibody to confirm Tspan14 protein expression in mouse and human platelets, and to demonstrate an association with ADAM10 in these cells. The best characterized ADAM10 substrate on platelets is the collagen receptor GPVI, which is emerging as a promising anti-platelet drug target for the treatment of arterial thrombosis (40). Interestingly, GPVI can be rapidly shed from the platelet surface following platelet activation, but is protected from ADAM10-mediated cleavage by an undefined mechanism (4, 5). Since we have shown that Tspan14 significantly reduces GPVI cleavage in a cell line model, it is possible that Tspan14 functions as the GPVI protector on resting platelets.

ADAM10 has both disease-promoting and disease-inhibiting activities, depending on the disease. Inhibition of ADAM10 activity could be beneficial for several diseases, in particular cancer, inflammatory diseases, asthma, and skin disorders (1). In contrast, promotion of ADAM10 activity on neurons could alleviate Alzheimer disease by preventing the generation of pathogenic β-amyloid peptides (1), and on platelets could prevent heart attack and stroke caused by thrombosis, through collagen receptor GPVI shedding (4, 5). Our data suggest that a future therapeutic strategy could be to target the LEL of a specific TspanC8 to disrupt its interaction with ADAM10. This could lead to ADAM10 activation, or inactivation, or internalization and degradation, and further research is required to investigate such possibilities. Nevertheless, such a therapeutic approach might modulate ADAM10 activity toward only certain substrates, and without the toxic side effects of targeting ADAM10 on every cell type in the body.

## Author Contributions

P. J. N. designed, performed, and analyzed the experiments shown in Figs. 2–9, designed the study and wrote the manuscript. J. Y. designed, performed, and analyzed the experiments shown in Figs. 2, 4, 5, and 7. JSR designed, performed, and analyzed the experiments shown in Figs. 1 and 10. ALM designed, performed, and analyzed the experiments shown in Fig. 10. A. E. C., J. F., and D. A. R. designed and generated expression constructs and gained preliminary data for Figs. 2, 4, 5, and 7. G. E. R. designed the experiments shown in Fig. 1 and helped to write the manuscript. MGT designed, performed, and analyzed the experiments shown in Fig. 1, designed and coordinated the study and wrote the manuscript. All authors reviewed the results and approved the final version of the manuscript.

## Footnotes

2 The abbreviations used are:

ADAM: A disintegrin and metalloprotease
ER: endoplasmic reticulum
APP: amyloid precursor protein
LEL: large extracellular loop
WGA: wheat germ agglutinin
HUVEC: human umbilical vein endothelial cells.
